# Characterization of Hematopoiesis in Sickle Cell Disease by Prospective Isolation of Stem and Progenitor Cells

**DOI:** 10.3390/cells9102159

**Published:** 2020-09-24

**Authors:** Seda S. Tolu, Kai Wang, Zi Yan, Shouping Zhang, Karl Roberts, Andrew S. Crouch, Gracy Sebastian, Mark Chaitowitz, Eric D. Fornari, Evan M. Schwechter, Joan Uehlinger, Deepa Manwani, Caterina P. Minniti, Eric E. Bouhassira

**Affiliations:** 1 Department of Medicine, Division of Hematology, Albert Einstein College of Medicine, Bronx, NY 10461, USA; stolu@montefiore.org (S.S.T.); Andrew.crouch@einstein.yu.edu (A.S.C.); grsebast@montefiore.org (G.S.); machaito@montefiore.org (M.C.); caterina.minniti@einsteinmed.org (C.P.M.); 2 Department of Cell Biology, Albert Einstein College of Medicine, Bronx, NY 10461, USA; kai.wang@tarabiosystems.com (K.W.); zi.yan@einstein.yu.edu (Z.Y.); shouping.zhang@einstein.yu.edu (S.Z.); karl.roberts@einsteinmed.org (K.R.); 3 Department of Orthopedic Surgery, Montefiore Medical Center, Bronx, NY 10461, USA; EFORNARI@montefiore.org (E.D.F.); ESCHWECH@montefiore.org (E.M.S.); 4 Department of Pathology, Division of Transfusion Medicine, Montefiore Health System, Bronx, NY 10467, USA; JUEHLING@montefiore.org; 5 Pediatric Hematology/Oncology/Marrow and Blood Cell Transplantation, Montefiore Health System, Bronx, NY 10467, USA; DMANWANI@montefiore.org

**Keywords:** sickle cell disease, hematopoietic stem cells, hydroxyurea, transfusion

## Abstract

The consequences of sickle cell disease (SCD) include ongoing hematopoietic stress, hemolysis, vascular damage, and effect of chronic therapies, such as blood transfusions and hydroxyurea, on hematopoietic stem and progenitor cell (HSPC) have been poorly characterized. We have quantified the frequencies of nine HSPC populations by flow cytometry in the peripheral blood of pediatric and adult patients, stratified by treatment and control cohorts. We observed broad differences between SCD patients and healthy controls. SCD is associated with 10 to 20-fold increase in CD34^dim^ cells, a two to five-fold increase in CD34^bright^ cells, a depletion in Megakaryocyte-Erythroid Progenitors, and an increase in hematopoietic stem cells, when compared to controls. SCD is also associated with abnormal expression of CD235a as well as high levels CD49f antigen expression. These findings were present to varying degrees in all patients with SCD, including those on chronic therapy and those who were therapy naive. HU treatment appeared to normalize many of these parameters. Chronic stress erythropoiesis and inflammation incited by SCD and HU therapy have long been suspected of causing premature aging of the hematopoietic system, and potentially increasing the risk of hematological malignancies. An important finding of this study was that the observed concentration of CD34^bright^ cells and of all the HSPCs decreased logarithmically with time of treatment with HU. This correlation was independent of age and specific to HU treatment. Although the number of circulating HSPCs is influenced by many parameters, our findings suggest that HU treatment may decrease premature aging and hematologic malignancy risk compared to the other therapeutic modalities in SCD.

## 1. Introduction

Hematopoiesis in patients with sickle ccell disease (SCD) is associated with erythroid hyperplasia, reticulocytosis, and ineffective erythropoiesis starting at the immature erythroblast stage in the bone marrow (BM) [1,2,3,4]. The consequences of SCD on stem and progenitor cells have not been fully characterized. Using a functional assay to characterize hematopoiesis, Hara et al. reported in 1977 that there were more burst forming unit-erythroid (BFU-E) cells circulating in the peripheral blood (PB) of patients with SCD compared to healthy individuals [5]. Croizat et al. expanded upon these results and observed increased numbers of BFU-Es, specifically in a subset of SCD patients with low HbF, but could not confirm the correlation between HbF levels and number of colony forming cells (CFCs) in a larger cohort [6,7]. In 2003, the number of circulating CD34^+^CD38^−^ HSPCs was shown to be slightly higher in steady state SCD patients as compared to controls, but dramatically higher during vaso-occlusive crises by Lamming et al. [8]. At the level of BM, Uchida et al. observed lower levels of CD34^+^ cells in SCD patients treated with HU as compared to “steady-state” or SCD patients without treatment [9], suggesting that some of the discrepancies in the previous reports might have been caused by the lack of stratification of the patients by treatment modality. 

The relative lack of data on the effect of ongoing hematopoietic stress, hemolysis, vascular damage, and chronic therapies, such as blood transfusions and hydroxyurea has become a pressing issue as patients with SCD now live longer and potentially myelotoxic therapies, such as HU, are initiated in younger populations, starting in children [10]. 

Hematopoiesis: In the classical model of hematopoiesis, long-term hematopoietic stem cells (LT-HSCs) reside at the apex of the hierarchy and differentiate into cells with progressively reduced self-renewal and differentiation potential [11,12]. 

In 2007, Majeti el al. reported that cord blood lin^−^, 34^+^, 90^+^, 38^−^, 45RA^−^ cells (hereafter referred to as HSCs) were enriched in LT-HSC activity [13]. In 2011, Notta et al. revealed that about one in 10 human cord blood cells that are lin^−^, 34^+^, 90^+^, 38^−^, 45RA^−^ also express the CD49f antigen (integrin-α6) and that these cells, hereafter referred to as 49f cells, have multi-lineage reconstitution potential for at least 20 weeks in a mouse xeno-transplantation assay [14]. Thus, significant progress has been made in identifying and quantifying LT-HSCs in cord blood.

Prospective quantification of adult human LT-HSCs has proven more difficult. Wang et al. [15] reported that about 1 in 400 adult BM 49f cells could engraft for at least 20 weeks. Huntsman et al. [16] reported frequencies of engraftment of 49f cells purified from mobilized peripheral blood (PB) as high as 1 in 50 at eight weeks after transplantation, but did not report on the long-term outcome. Using a novel somatic transversion capture/recapture approach, Wang et al. reported in 2020 that adult 49f cells could produce both myeloid and lymphoid cells for at least three years in adults [17].

Data on the phenotype of long-term repopulating cells based on clinical transplantation are also very sparse. There is strong evidence that the lin^−^, 34^+^, 90^+^, 38^−^, 45RA^−^ fraction contains most engraftable adult cells but the minimum number of cells necessary for engraftment is not well defined [18]. 

The differentiation stage immediately after the LT-HSCs is under considerable debate [19,20,21,22], but most authors agree that at least a fraction of LT-HSCs become multipotent progenitors (MPP). MPP are defined as cells that are able to transiently produce lympho-myeloid output after transplantation in immuno-deficient mice. Once MPPs have lost expression of CD90, they become lin^−^, 34^+^, 90^−^, 38^−^, 45RA^−^ [13]. 

In turn, MPPs differentiate into more committed progenitors that can be identified using CD38 and a combination of other markers. The Common Myeloid Progenitors (CMPs, Lin^−^ 34^+^ 38^+^ 123^+^ 45RA^−^), Megakaryocyte-Erythroid Progenitors (MEPs, Lin^−^ 34^+^ 38^+^ 123^−^ 45RA^−^) and Granulocyte Macrophage Progenitors (GMPs, Lin^−^ 34^+^ 38^+^ 123^+^ 45RA^+^) [23,24] are the three classical myeloid progenitors. CMPs were initially believed to be a homogeneous population that was the source of all myeloid cells downstream of MPPs and upstream of GMPs and MEPs. However, this scheme has been questioned because of the identification of lymphoid-primed multi-potential progenitors (LMPPs, Lin^−^ 34^+^ 38^−^ 45RA^+^ 90^−^), which do not express CD38 but express CD45RA, and have lymphoid and granulocyte−macrophage but no megakaryocyte−erythroid potential [25]. In addition, the CMP cell fraction has recently been shown to be heterogeneous and composed of two populations, including one which gives rise to erythroid cells [26]. 

Despite this complexity and remaining uncertainties, this body of knowledge provides a set of tools to prospectively isolate and quantify HPSCs which have seldom been exploited in the context of SCD. 

In order to gain insight into the consequences of SCD on the hematopoietic system, we have quantified the frequencies of nine HSPC populations defined by flow cytometry in the PB and in selected BM of SCD individuals that are stratified by three treatment modalities: naïve, chronic transfusion therapy, and long term HU therapy. 

## 2. Materials and Methods

### 2.1. IRB Approval

All samples were acquired under protocols approved by the Internal Review Board of the Albert Einstein College of Medicine (IRB # 2017-8034, initial approval date: 11/20/2017) 

### 2.2. Sample Acquisition

Clinical and laboratory data from over 1000 patients with SCD were collected and reviewed from Montefiore Medical Center’s adult and pediatric clinics. From this population, 70 patients were selected for the study based on genotype, sickle cell anemia (SCA): HbSS and HbSB^0^; willingness to provide consent and clinical criteria. These criteria were defined by clinical stability which included lack of an active vaso-occlusive event or pain crisis in the past two weeks, non-pregnant state, and no active underlying malignancy as presence of any these factors would lead to confounding by altering the proportion of hematopoietic stem cells in the peripheral blood.

These 70 patients were stratified as either on HU (defined as having received HU for a minimum of 3 months), chronic transfusion therapy (receiving current monthly exchange or simple transfusions ongoing for minimum of one year), or treatment naïve (not on any SCD specific modifying therapy). 

Ten to twenty mL of PB were then collected from each patient at steady state in yellow top tubes and processed within 4 to 16 h of collection. Steady state was defined as free of clinical complications, including vaso-occlusive complications, or any acute exacerbations for a minimum of 3 weeks. Samples from the transfusion group were obtained just prior to the next transfusion (which was generally 4 to 6 weeks from the previous time of transfusion). 

All patients treated with HU were prescribed a stable dose of HU for at least 3 months prior to PB collection. Average years on HU in this cohort were 7.7 ± 5.95 with average dose of 1361.65 ± 445.15 mg (19.27 ± 5.31 kg/mg)

In addition, 5 of the 70 patients (all in the HU group) had paired BM aspirates obtained while undergoing hip core decompression or hip arthroplasty. As controls, 10 BM aspirates and paired PB from healthy African Americans, age 18 to 45, were purchased from Lonza (Basel, Switzerland). Ten additional PB samples from healthy African American volunteers, age 18 to 30, were collected at Albert Einstein College of Medicine. 

Complete blood counts were performed using a Sysmex XN−9000 hematological analyzer.

Mononuclear cells were isolated using Histopaque as recommended by the manufacturer (Sigma−Aldrich, St Louis, MD, USA) and aliquots were frozen in liquid nitrogen. 

Plasma was collected by centrifugation and stored in liquid nitrogen for further processing. 

### 2.3. FACS Analysis

FACS analysis was performed on a Cytek Aurora using the antibody panel described in Table S1. 

Cells were first gated based on forward and side scatter to eliminate debris and doublets. Dead cells were filtered using a Zombie dye (Zombie−NIR, Biolegends, San Diego, CA, USA). Data were analyzed using FloJo 10.6 (Ashland, OR, USA). The positioning of each gate was established using concatenated FCS files representing 20 different samples. Once established, the gates were automatically applied to each sample individually. 

The lineage markers mix included CD2, CD3, CD4, CD7, CD8, CD10, CD14, CD19, CD20, CD56, and CD235a. CD235a was labeled with FITC; all other lineage markers with PE−Cyanine5.

Whenever possible, a million cells were acquired to be able to quantify rare populations. Samples with less than 50,000 live cells (because of small amount of blood collection or low recovery after thawing and staining) were eliminated from the analysis.

### 2.4. Data Analysis

The number of CD34^+^ cells and HSPCs per µL of PB was calculated by dividing the number of live cells observed by FACS by the μL of blood analyzed. The μL of blood analyzed was estimated using the total number of cells analyzed by FACS, the percentage of mononuclear cells in the blood, and the white blood cell count. The latter two values were obtained using a hematological analyzer. Five out of the 70 peripheral blood samples were excluded in data analysis due to insufficient quantity required to run accurate flow cytometry.

### 2.5. Statistical Analysis

All analysis were performed using R 3.6.3. Regression analysis was performed using the glm R package.

Graphs and *p*-values were produced using the ggplot2 package. Since the data did not follow a Gaussian distribution, we compared the means using non-parametric Wilcox test. 

Correction for multiple testing was performed using the false discovery rate (FDR) approach [27]. 

Significance levels are encoded as follows: ns: *p* > 0.05; *: *p* ≤ 0.05; **: *p* ≤ 0.01; ***: *p* ≤ 0.001, ****: *p* ≤ 0.0001.

In Figure 3, the dot plots are standard R’s default boxplots. 

The box outlines the first and third quartiles. 

The horizontal bar represents the median; the diamond represents the mean. 

The whiskers are calculated as follows: Upper whisker = min(max(x), Q_3 + 1.5 * IQR); lower whisker = max(min(x), Q_1 − 1.5 * IQR), where IQR = Q_3 − Q_1, the box length. 

In summary, the upper whisker is located at the “smaller” of the maximum x value and Q_3 + 1.5 IQR, whereas the lower whisker is located at the “larger” of the smallest x value and Q_1 − 1.5 IQR. 

## 3. Results

Clinical and laboratory characteristics of the study population are described in Table 1. Analysis of hematological and red blood cells (RBC) parameters revealed that the HU treated group had significantly higher levels of HbF, mean corpuscular volume (MCV), erythropoietin (EPO), and platelets compared to the naive and transfusion groups, while the transfusion group had significantly higher levels of bilirubin and ferritin compared to the HU group (Table 1 and Figure S1).

These results were consistent with expectations based on treatment type, and importantly, confirmed medication adherence in the HU group with average time on HU of 7.7 ± 5.95 years and average dose of 1361.65 ± 445.15 mg (19.27 ± 5.31 kg/mg).

**Figure 1:**
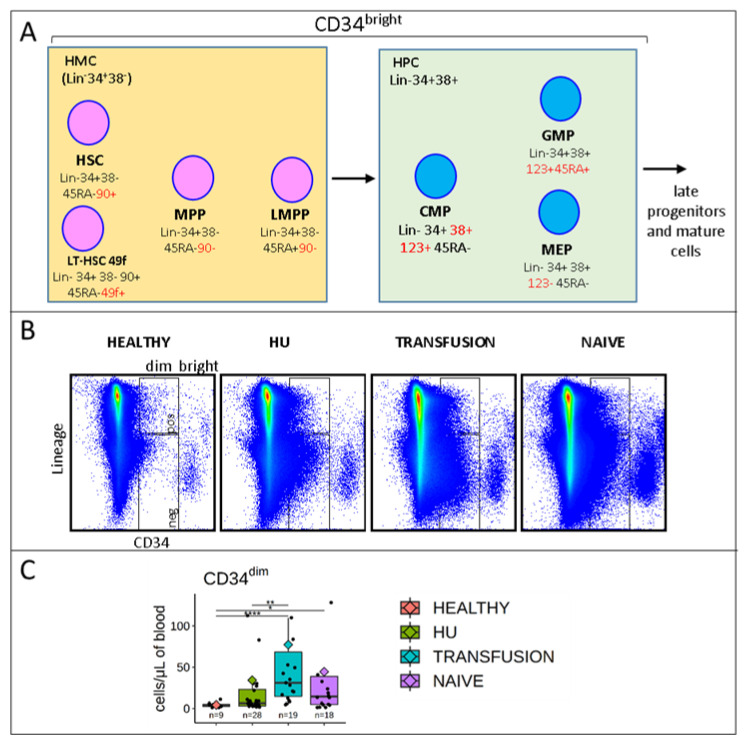
CD34^dim^ cells are elevated in sickle cell disease (SCD): (**A**) Diagram illustrating the 9 populations of hematopoietic stem and progenitor cells (HSPCs) quantified in this report. The Hematopoietic Multipotent Cell (HMC) population encompasses 49f cells, 90^+^45RA^−^, MPPs, and LMPPs; the Hematopoietic Progenitor Cell (HPC) population includes the Common Myeloid Progenitors (CMPs), Granulocyte Macrophage Progenitors (GMPs), and Megakaryocyte-Erythroid Progenitors (MEPs). HMCs and HPCs differ in part by expression of CD38. (**B**) High number of CD34^dim^ cells in SCD patients: Dot plots illustrating the flow cytometry assays. (Healthy, HU = Hydroxyurea treated, Transfusion = treated by chronic transfusion, or Naïve = treatment free). FCS files were concatenated (10,000 cells per patient) and plotted to visualize expression of CD34 and lineage antigens. (**C**) Boxplots summarizing the concentration of CD34^dim^ cells per μL of blood. The box outlines the first and third quartiles. The horizontal bar represents the median; the diamond represents the mean. Significance was calculated using the Wilcox test (with FDR correction). ns: *p* > 0.05; * *p* ≤ 0.05; ** *p* ≤ 0.01; **** *p* ≤ 0.0001. CD34^dim^ cells are elevated in SCD.

CD34^dim^ cells are highly elevated in SCD patients: To characterize hematopoiesis in our cohorts of patients, we first focused on the CD34 expressing cells. As expected, there was a well-defined population of CD34^bright^ cells in all individuals, which contains the HSPCs and a more diffuse population of CD34^dim^ cells (Figure 1A,B). 

Analysis of the latter population revealed that there were 10 to 20 fold more CD34^dim^/μL of blood in HU, transfusion, and naive patients than in healthy controls (34.1 ± 15.2, 77.1 ± 26.7, and 44.4 ± 14.7 vs. 4.16 ± 1.01 CD34^dim^ cells/μL of blood) (Figure 1C). 

The viability of the CD34^dim^ and CD34^bright^ cells was similar but the vast majority of the CD34^bright^ cells were within a narrow FSC and SSC gate, while the CD34^dim^ had more variable scatter properties (Figure S2A). 

Analysis of surface antigens revealed that in both SCD patients and controls, the CD34^dim^ population was characterized by a complex mixture of cells expressing high levels of CD49f and CD123, and variable levels of lineage, CD90, CD45RA, CD38, CD33, and CD235a antigen (Figure S2B). Although the functional properties of these CD34^dim^ cells are not known, these observations suggest that this hematopoietic compartment is deeply disturbed in SCD patients, particularly in the transfusion group where virtually all patients had elevated levels of CD34^dim^ cells.

CD34^bright^ cells are significantly elevated in the PB of SCD patients in the transfusion and naive group, but not in the HU group: 

The CD34^bright^ cells had a broad range of concentrations across all three groups of SCD patients (from about 1 to >50/μL), reflecting heterogeneity within each group. The 28 HU patients analyzed had an average (± S.E.M) of 6.3 ± 1.3 CD34 ^bright^/μL of blood which was higher, though non-significantly, than the 2.5 ± 0.5 CD34 ^bright^/μL observed in 19 healthy controls.

**Figure 2:**
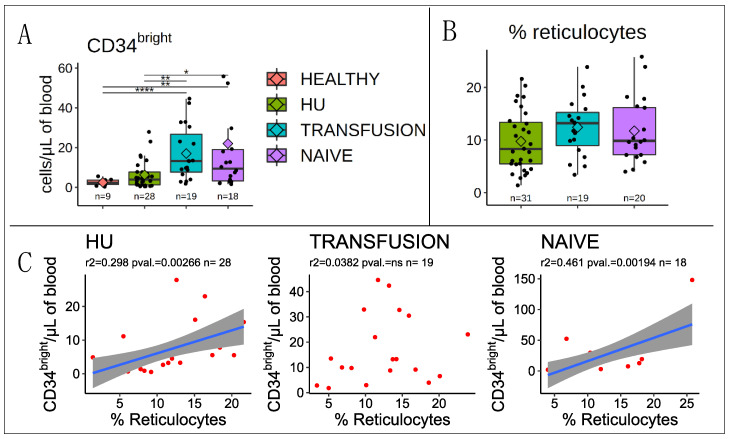
CD34^bright^ cells are elevated in transfusion and naive groups: (**A**) CD34^bright^ cells: Boxplot illustrates the concentration of CD34^bright^ cells per mL of blood. Boxplots and significance are as in Figure 1. All three groups of SCD patients exhibit higher mean concentration of CD34^bright^ than healthy individuals, but in the HU group, the average concentration of CD34^bright^ cells is lower than in the other two groups and much closer to that of healthy individuals. (**B**) Boxplot illustrates the percentage of reticulocytes in patients with SCD. The reticulocyte percentages in all three groups of SCD patients averages 10 to 15%, much higher than the normal range, suggesting that regardless of treatment, erythropoiesis is not completely normalized and is higher in SCD patients than in healthy individuals. (**C**) Plots illustrating a regression analysis between the concentration of CD34^bright^ cells in the blood and the percentage of reticulocytes. * *p* ≤ 0.05; ** *p* ≤ 0.01; **** *p* ≤ 0.0001.

By contrast, the 20 naive and 19 transfusion patients examined respectively exhibited 13.6 ± 3.1 and 22.0 ± 8.3 CD34 ^bright^/μL of blood which was significantly higher than the healthy controls (*p*-values <0.01 in all cases) (Figure 2A). This was true whether the data was normalized to the number of µL of blood or to the number of live mononuclear cells. 

Therefore, treatment with HU normalized the number of circulating CD34^bright^ cells in SCD patients to levels similar to that of healthy controls. 

### 3.1. Correlation with Reticulocytes

Average reticulocyte levels were about 10−15% in all three SCD groups, suggesting that none of the treatments were sufficient to restore a normal rate of RBC production in most patients (Figure 2B). Since the reticulocytes levels in the transfusion group were measured just prior to transfusion, they were likely at their highest level at the time of measurement (see discussion).

Importantly, we observed a strong correlation between CD34^bright^ cells and percent reticulocytes in the naïve cohort, a weaker correlation in HU-treated cohort, and no correlation in the transfusion cohort, suggesting that in naive patients the increase in circulating CD34^bright^ cells reflects increased RBC production. However, this association is lost in the transfusion group (Figure 2C). 

### 3.2. Stem and Progenitor Cells Quantification

**Figure 3:**
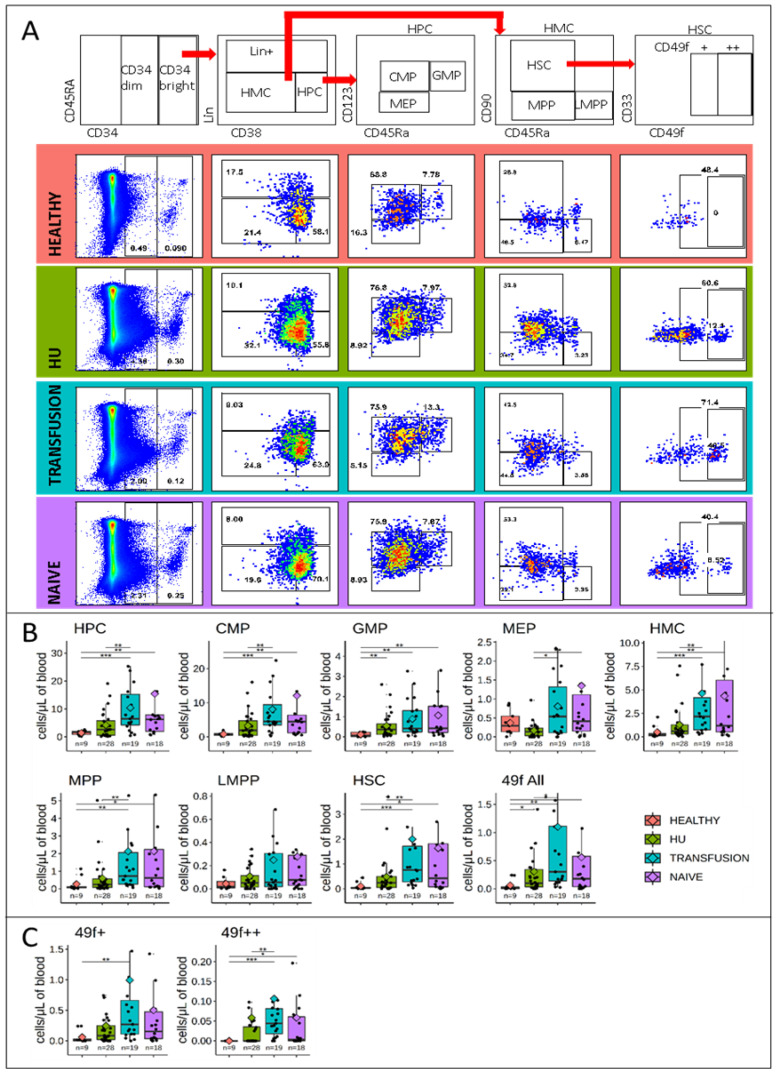
HSPCs characterization: (**A**) Sorting scheme and dot plots illustrating the FACS analysis. Each dot plot was generated by concatenating the FACS data obtained from all patients in the group. (**B**,**C**) Concentrations of HSPCs per mL of blood. Boxplots illustrate the concentration of 9 sub-populations of HSPCs in the three groups of SCD patients and in controls. Boxplots and significance are as in Figure 1. All populations except MEPs and LMPPs are in higher concentration in the SCD patients, but the HU-treated patients are closer to the controls than the other two SCD groups, suggesting that treatment with HU partially normalizes the circulating HSPC concentrations. * *p* ≤ 0.05; ** *p* ≤ 0.01; *** *p* ≤ 0.001.

In order to gain additional insight regarding hematopoiesis in SCD, we quantified the 9 populations depicted in Figure 1A. These populations include the myeloid progenitor compartment defined as a population of Hematopoietic Progenitor Cells (HPCs, Lin^−^CD34^+^CD38^−^) which encompasses the CMPs, GMPs, MEPs, and the stem cell compartment defined as a population of Hematopoietic Multipotent Cells (HMCs, Lin^−^CD34^+^CD38^+^), which encompasses the 49f, HSCs, MPPs, and LMPPs. The gating strategy to isolate these various cell populations is described in Figure 3A.

To verify that we could reproducibly quantify these populations of cells in the unstimulated PB, we analyzed technical duplicates which demonstrated that our measurements were highly reproducible (*r*^2^ > 0.9 in most cases, Figure S3).

FACs analysis revealed that the concentration of HMCs, MPPs, HSCs, 49f cells, HPCs, CMPs, and GMPs per μL of blood followed the same trend as that of the bulk CD34^bright^ population, with the lowest levels in controls, a slight elevation in HU patients, and higher levels in transfusion and naive patients (Figure 3A,B). By contrast, the proportion of MEPs was two-fold lower in the HU group (*p*-value < 0.05) and not significantly different between the healthy, naïve, and transfusion groups. The concentration of LMPPs, which was generally very low, was similar in all groups. 

### 3.3. CD235a is Expressed in a Fraction of PB HMCs, MPPs, and HSCs of SCD Patients but not in Controls

CD235a (glycophorin A), a marker that is upregulated during erythroid differentiation is generally not expressed in the stem cell compartment of healthy individuals but it has previously been reported that SCD patients harbor a population of CD34^+^CD38^−^ cells that co-express this marker [28].

**Figure 4:**
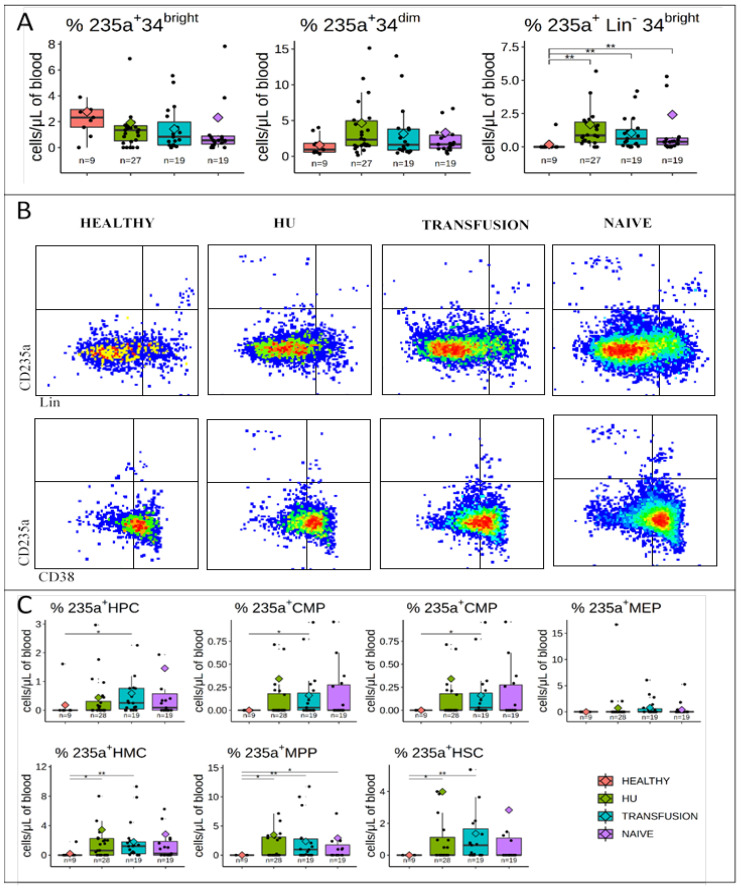
CD235a is over expressed in HMCs, MPPs, and HSCs of SCD patients: (**A**) Boxplots illustrating expression of CD235a on CD34^bright^, CD34^dim^ and lin^−^CD34^bright^ cells. Boxplots and significance levels are as in Figure 1. The fraction of cells expressing CD235a is similar in bulk populations of CD34^bright^ and CD34^dim^ cells in controls and in SCD patients, but is much higher on the lin^−^34^bright^ cells of the SCD patients. (**B**) Concatenated dot plots illustrating expression of CD235a as a function of lineage antigens and CD38 expression. (**C**) Boxplots summarizing the percentages of HSPCs expressing CD235a. CD235a is expressed in a significantly higher fraction of HMCs, MPPs, and HSCs in SCD patients than in controls. 49f expressing cells and LMPP could not be analyzed for 235a expression due to the low frequency of these populations. * *p* ≤ 0.05; ** *p* ≤ 0.01.

To define more precisely the type of HSPCs that express CD235a, we analyzed its expression in defined populations. Initial analysis revealed that about 2 to 4% of the bulk CD34^bright^ and CD34^dim^ cells expressed CD235a, but there was no difference between the healthy and the three SCD treatment groups (Figure 4A). 

Examination of additional markers revealed that there was almost no CD235a expression on Lin^−^CD34^bright^ cells of the healthy individuals in contrast to expression in 2 to 3% observed in SCD patients (Figure 4A). 

Stratification of the Lin^−^CD34^bright^ into defined populations of HSPCs revealed that CD235a was highly over-expressed in the HMCs, MPPs, and HSCs of SCD patients in all three treatment groups and somewhat less so in the HPCs, CMPs, and GMPs (Figure 4B,C). Importantly, expression of CD235a was highly variable across all three treatment modalities, with some patients exhibiting undetectable levels of CD235a while others expressed highly variable levels, reaching more than 40% in some individuals. 

These results confirm and expand upon the previous observation that expression of the erythroid marker, CD235a, is altered in the stem cell compartment of a fraction of SCD patients.

### 3.4. CD49f is Expressed at Very High Levels in a Subset of HSCs from SCD Patients

Expression of the 49f cell antigen (integrin-α6) on HSCs in healthy controls does not form a distinct population (Figure 3B). Rather, its expression is continuous and the gate to define the 49f cells is determined based on the location of negative controls. Importantly, SCD patients exhibited a population of Lin^−^CD34^bright^CD38^−^CD45RA^−^CD90^+^ that expresses very high levels of the 49f antigen which was almost absent in the healthy controls. We named this population, 49f++ cells. Concentration of the CD49f++ cells were 0.058 ± 0.03; 0.1 ± 0.04, and 0.057 ± 0.03/μL of blood (mean ± SE) for the HU, transfusion, and naive groups, respectively. 

The nature of these cells is unclear, but their presence strongly affects the quantification of the HSCs and LT−HSCs in SCD patients.

### 3.5. MPPs and HSCs and 49f Cells are Particularly Abundant in SCD

**Figure 5:**
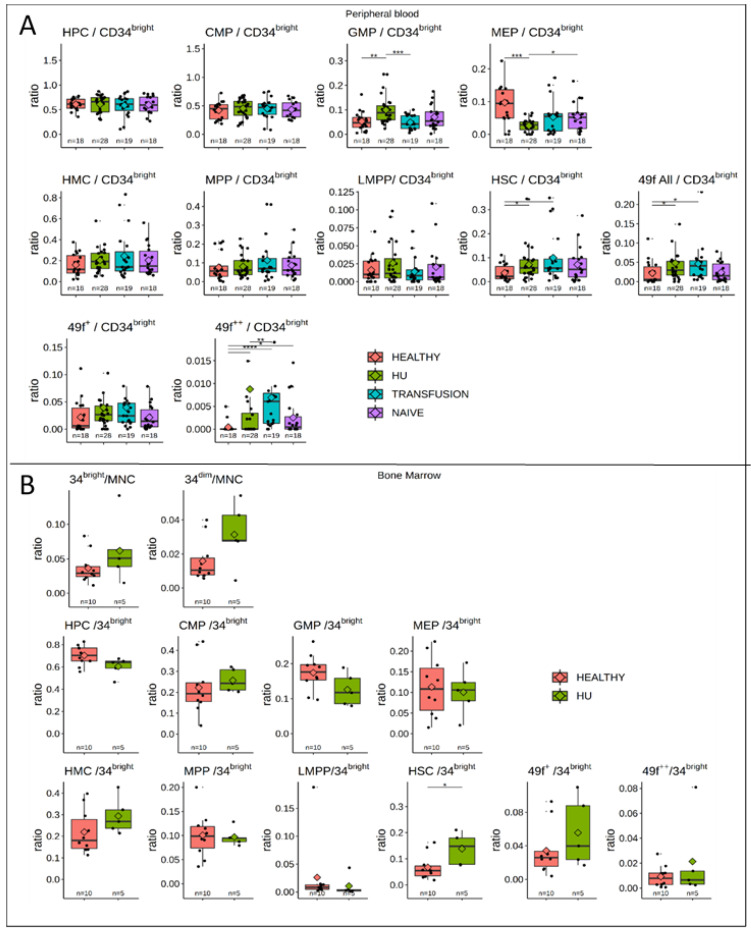
SCD patients are depleted in circulating MEPs but exhibit higher frequencies of HSCs and 49f cells: (**A**) Boxplots illustrating the proportion of HSPCs in PB relative to the number of CD34^bright^ cells. Boxplots and significance are as in Figure 1. The proportion of circulating MEPs/CD34^bright^ cells is lower in all SCD patients than in controls, but the decrease is more pronounced in HU patients. By contrast, the proportions of HSCs and 49f cells/CD34^bright^ cells is higher in HU and transfusion treated patients than in controls and naive individuals. Some, but not all, of the increase in HSC and 49f cells is caused by the increased proportion of 49f++ cells. (**B**) BM analysis: BM from 10 controls and 5 SCD patients treated with HU were analyzed by FACS using the same protocol as the PB cells. The number of CD34^bright^/MNCs is higher in SCD patients, but the difference does not reach significance. None of the concentration of HSPCs relative to the CD34^bright^ cells, except for HSCs, is significantly different between the SCD patients and the controls. * *p* ≤ 0.05; ** *p* ≤ 0.01; *** *p* ≤ 0.001, **** *p* ≤ 0.0001.

To directly compare the relative proportion of the various HSPCs to one another, we normalized the number of HSPCs to the total number of circulating CD34^bright^ cells, as reported by others [29]. This revealed two major differences between SCD patients and controls (Figure 5). First, MEPs which represent 9.6 ± 1.8% of the CD34^+^ cells in the healthy controls, were significantly lower in SCD patients with averages of 5.2 ± 0.1% and 5.4 ± 0.1% in the transfusion and naive groups, respectively, and only 2.6 ± 0.4% in the HU group. 

Second, the number of HSCs observed in the healthy controls was 3.7 ± 0.8%, which was significantly lower than the SCD groups which exhibited averages of 7.1 ± 1.3%, 10.0 ± 2.3% and 7.1 ± 1.7% for the HU transfusion, and naive cohorts, respectively. 

This increase in the percentage of HSCs was also detected in the 49f expressing cells with average of 0.08 ± 0.06% in the healthy control versus 5.1 ± 1.1%, 5.5 ± 1.3%, and 3.5 ± 1.0% in the HU, transfusion, and naive cohorts respectively. 

Sub-dividing the 49f cells into 49f+ and 49f++ cells revealed that both cell populations were increased in the HU and transfusion groups, although the CD49f++ cells contributed the most to this increase.

### 3.6. Bone Marrow Analysis

Differences in the concentration of HSPCs in the peripheral blood can reflect changes in trafficking between the BM and the circulation or changes in hematopoietic activity within the BM.

To assess changes in bone marrow activity, we compared five BM samples from patients treated with HU with 10 healthy control BMs (Table 2). In this small cohort, the levels of HSCs were higher in the SCD than in the healthy groups (*p* = 0.033), while the number of CD34^bright^ CD34^dim^ cells, and all other HSPCs, were similar (Figure 5B).

To determine if the patterns we observed in the PB reflect frequencies of HSPCs in the BM, we also compared eight paired PB and BM control samples. This revealed that HSPCs frequencies in the PB and BM were generally well correlated (*r*^2^ = 0.45−0.65, Figure S4), suggesting that results obtained in the PB reflect, in part, changes in the BM.

### 3.7. Number of Circulating HSPCs Decreases as a Function of the Duration of HU Treatment

**Figure 6:**
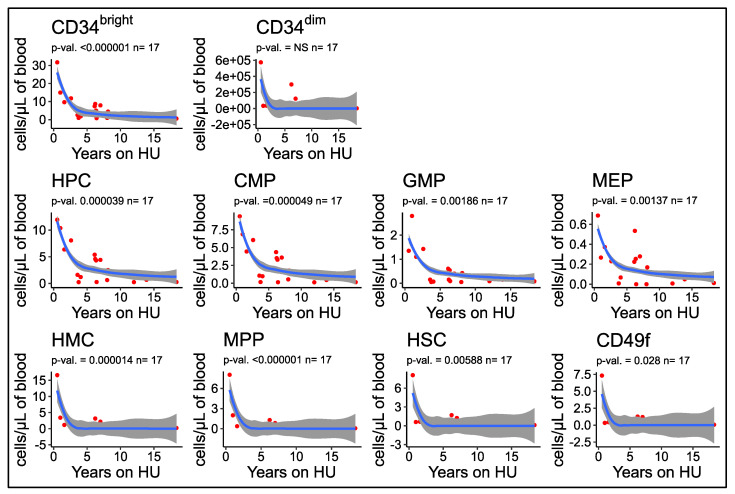
Concentrations of HPSCs decrease as a function of length of HU treatment. Plots illustrating the concentration of HSPCs/μL of blood as a function of the length of HU treatment. Data was fitted using a power function and p-values were calculated using the R glm package. The concentrations of CD34^bright^ and HSPCs decrease with length of time of HU treatment. The concentration of CD34^dim^ cells does not exhibit any significant time dependency. Red curve represents the best fit line for the power model (y ~ log(x)). (*p*-values for the model are provided above). Grey smooths represent the 95% confidence interval of the power model.

Importantly, analysis of the relationship between the concentration of circulating HSPCs and years of HU treatment revealed an inverse correlation between these two parameters. Curve fitting analysis revealed that the relationship was not linear since the best fits were observed using power, or exponential time decay curves (Figure 6) which suggested that the concentrations of CD34^bright^ cells and HSPCs decrease exponentially over time in patients treated with HU.

Analysis after log transformation of the time on HU yielded r2 values in the 0.7 to 0.5 range for CD34^bright^ cells and for subtypes of HPCs, and lower correlations with the CD34^dim^ and the HMC populations (Figure S5). Additional analyses revealed that there was no correlation between the concentrations of HSPCs in the blood and the age of the patients in the HU group or in the other treatment groups (data not shown).

## 4. Discussion

In this study we report on the composition of HSC in patients with SCD under different clinical conditions: Naïve, on chronic transfusion and on HU. We demonstrate that the concentration per volume of blood of CD34^bright^ cells is significantly higher (*p* < 0.01) in patients on chronic transfusion or naive, but almost unchanged in the HU group compared to the healthy controls. Analysis of sub-populations revealed that, except for MEPs, this finding extended to most populations of HSPCs which were significantly higher in naive and transfused patients but much less so in HU-treated patients. Normalizing the data to the total number of CD34^bright^ demonstrated a specific depletion of MEPs in the HU group and an over-representation of HSCs and 49f cells in both the HU and transfusion groups. Uchida et al. previously reported that HU treated patients had lower BM and PB concentrations of CD34^+^ cells, and lower numbers of colony forming progenitors compared to patients not treated with HU, suggesting that treatment with HU either slowed hematopoiesis and/or decreased the number of circulating cells [9]. Here, we confirmed and expanded these results to nine populations of HSPCs and three treatment groups. 

CD34^+^ expressing cells could be divided into a homogeneous CD34^bright^ population easily differentiated from the negative cells, and a more diffuse CD34^dim^ population located between the negative and the CD34^bright^ cells. These CD34^dim^ cells were present at concentrations that were 10-fold higher in the HU and naive groups and twenty-fold higher in the transfusion group as compared to the healthy controls. 

Leonard et al. [1] recently observed significantly more CD34^dim^ cells in the BM of SCD patients as compared to healthy controls and suggested that these CD34^dim^ cells might be erythroid progenitors because cord blood lin^−^CD34^low^ had previously been shown to be enriched in erythroid colony-forming cells [30]. Whether the CD34^dim^ cells that we observed in the PB of SCD patients are mostly erythroid progenitors is unclear because most of the cells did not express the CD38 antigen while the Lin^−^ CD34^low^ cord blood erythroid progenitors mentioned above were 95% CD38 positive. The most striking characteristic of the PB CD34^dim^ cells in our cohort were very high level expression of CD49f and CD123, which are not expressed at high levels in normal erythroid progenitors. Together, these observations demonstrate that this hematopoietic sub-compartment is deeply perturbed in SCD, underscoring the need for further investigation

Therefore, while the overall number of CD34^bright^ cells and HSPCs is partially normalized in the HU group, all SCD patients exhibit abnormal hematopoiesis, which is in accordance with the observation that none of the treatment modalities eliminate anemia and the resultant demand for RBC production as demonstrated by the high reticulocyte percentage in all three SCD groups. This chronic demand for increased RBC production seems to be met by increased differentiation of MEPs, which is not compensated for by increased production of CMPs, and is associated with higher number of stem cells. 

In the classical model of hematopoiesis, this observation might be explained by the hypothesis that chronic anemia results in the accumulation of HSCs and 49f cells that do not differentiate efficiently into CMPs to meet the demand for MEPs. However, this classical model has been questioned. For instance, it has been proposed that a small population of CD34 negative cells are the true adult stem cells and that these cells can directly differentiate into MEPs [31]. Under this model, the increase in HSCs in SCD patients might be a direct response to the MEP depletion. 

The concentration of CD34^bright^ cells was correlated with the reticulocyte percentage in the naive and HU groups, but not in the transfusion group. The reticulocyte percentage is a complex cyclical biomarker in SCD patients that is affected by the time since the last transfusion, oxygen delivery, and blood viscosity [32,33,34]. In our cohort of chronically transfused patients, the reticulocyte counts were likely at maximal levels, since they were measured immediately prior to transfusion (hence, 4 to 6 weeks after the last transfusion). The elevation in the number of most HSPCs and the lack of correlation between reticulocyte count and CD34^bright^ cells (and any of the other HSPC subpopulation, data not shown) in the transfusion group suggests that transfusions disrupt all hematopoietic compartments and each compartment may returns toward equilibrium with different kinetics in the interval between transfusions. 

Evidence of disrupted hematopoiesis was observed by analysis of CD235a and CD49f expression. Ectopic expression of CD235a on CD34^+^CD38^−^ cells was first reported by the Punam group [28] and confirmed by the Tisdale group [1] who demonstrated that expression of this antigen on HSPCs was not a technical artifact caused by adhesion of RBCs to the HSPCs. Here, we show that ectopic expression of CD235a is highly heterogeneous in all three groups of SCD patients and that it can be detected in an average of 2−5% of both MPPs and HSCs, reaching up to 40% in some individuals. As suggested by Luck et al. [28], cells prematurely expressing CD235a might be stress HSPCs primed to differentiate into the erythroid lineage. Alternatively, the CD235a HSPCs in SCD patients might be cells that are adopting a developmentally immature phenotype in response to anemia since CD235a has been reported by several investigators to be expressed on HSPCs differentiated from iPSCs, which are similar to embryonic or fetal HSPCs [35,36]. 

We detected a novel population of phenotypic HSCs that expressed CD49f at higher levels than healthy controls. This is of interest because integrin-α6, in addition to being expressed on cord blood LT−HSCs, has been shown to be expressed on multiple other types of human stem cells and may play a key role in maintaining stem cells in their niche [37]. The presence of 49f++ cells has not been detected before in human primary samples, but Fares et al. reported that cord blood CD34^+^ cells expanded in culture can differentiate into cells that expressed high levels of CD49f [38]. These cells, which had an immunophenotype identical to the 49f++ cells that we observed, were not engraftable. Hence, the 49f++ cells in the SCD patients might not be stem cells. As discussed above, the CD34^dim^ cells that are present in high amounts in SCD patients also express very high levels of the CD49f antigen. The CD34^bright^CD49f++ that we observed might, therefore, be the tail-end of this population. Understanding the functional characteristics of PB cells expressing high levels of CD49f is critical, as their presence interferes with the quantification of HSPCs in SCD patients. A recent report based on single cell sequencing revealed that a high percentage of CD34^+^ cells in BM harvested from children with SCD were B-lymphoid progenitor cells, thus reducing the proportion of other HSPC populations within the CD34 compartment [39]. Together with our studies, this report suggests that better biomarker are necessary for quantifying HSCs in SCD, which is essential for optimization of curative therapies.

Chronic stress erythropoiesis and inflammation caused by SCD and exposure to HU, an anti-metabolite chemotherapeutic, has long been suspected of causing premature aging of the hematopoietic system, and of increasing the risk of hematological malignancies [40,41]. An important observation that came out of our study is that we found that the concentration of CD34^bright^ cells and of all the HSPCs decreased logarithmically with time of treatment with HU. This correlation was specific to HU treatment and independent of age. 

The number of human HSCs increases with age, but older HSCs have decreased self-renewal capacity, poor reconstitution potential after transplantation, and exhibit a myeloid bias [42,43]. In pathological situations, the number of CD34^+^ and of HSPCs can be either lower, as in Fanconi anemia [44], unchanged, as in low risk BM failure MDS, or increased, as in high risk MDS and in myelofibrosis [29,45,46]. Given this wide spectrum of variation in the number of HSPCs in normal and pathological situations, interpreting the increased number of CD34^bright^ and HSPCs in SCD patients is inherently difficult. However, those exposed to HU exhibited normalization of HSPCs compared to patients on transfusion or naïve, which approached levels of normal controls. This observation is corroborated by a time dependent negative correlation of HSPCs with length of HU treatment. If these results are confirmed, one can speculate that HU treatment may be protective against premature aging on the stem cell compartment and decrease hematological malignancy risk. 

The characterization of HSPCs in SCD patients is important in order to define the best protocol to harvest cells prior to curative gene therapy and select the patients least likely to be subject to graft failure or to develop myelodysplastic syndrome (MDS) secondary to poor hematopoietic cell quality. The multiple differences that we have observed between controls and the three groups of SCD patients are the first steps to provide a foundation for the development of successful gene therapy protocols and may inform decision making among clinicians when treating patients with sickle cell disease. The future direction of this study will be to confirm these results in larger cohort of bone marrow samples and in functional assays.

## Figures and Tables

**Table 1:** Clinical and laboratory characteristics.

	All Patients			Hydroxyurea ∞			Transfusion α			Naïve ¥		
	Total	Adult	Pediatric	Total	Adult	Pediatric	Total	Adult	Pediatric	Total	Adult	Pediatric
**count**	71	44	27	32	21	11	19	10	9	20	13	7
**Age**	26.11 ± 12.24	33.09 ± 10.21	14.31 ± 2.25	28.06 ± 13.67	35.05 ± 11.06	13.4 ± 7.3	21.11 ± 7.3	26.7 ± 5.39	14.89 ± 2.64	27.85 ± 12.3	34.85 ± 9.6	14.86 ± 1.12
**Sex**	M = 36 F = 34	M = 22 F = 22	M = 14 F = 13	M = 16 F = 15	M = 11 F = 10	M = 5 F = 5	M = 12 F = 7	M = 7 F = 3	M = 5 F = 4	M = 8 F = 12	M = 4 F = 9	M = 4 F = 3
**Hb g/dL**	9.13 ± 1.37	9.27 ± 1.43	8.9 ± 1.22	9.5 ± 1.24	9.53 ± 1.37	9.43 ± 0.99	9.39 ± 0.99	9.66 ± 0.9	9.09 ± 0.99	8.33 ± 1.51	8.55 ± 1.58	7.9 ± 1.28
**Hb S %**	64.77 ± 23.59	66.84 ± 22.37	61.27 ± 25.12	74.3 ± 11.46	75.84 ± 9.28	71.06 ± 12.14	33.2 ± 12.14	34.73 ± 12.67	31.5 ± 11.29	80 ± 16.86	77.01 ± 20.11	85.54 ± 3.68
**Hb F%**	10.91 ± 7.6	10.77 ± 8.58	11.16 ± 5.57	16.24 ± 6.94	16.67 ± 7.43	15.34 ± 4.26	5.87 ± 4.26	3.07 ± 2.03	8.98 ± 3.91	7.44 ± 5.48	7.14 ± 6.45	7.98 ± 2.87
**Hematocrit %**	26.63 ± 3.78	26.92 ± 3.98	26.15 ± 3.37	27.03 ± 3.55	27.06 ± 3.75	26.97 ± 2.62	28.08 ± 2.62	28.71 ± 2.53	27.39 ± 2.54	24.64 ± 4.22	25.32 ± 4.57	23.37 ± 3.11
**MCV fL**	92.49 ± 11.22	93.7 ± 11.77	90.43 ± 9.87	101.38 ± 8.76	102.79 ± 9.31	98.44 ± 3.75	88.25 ± 3.75	86.47 ± 3.18	90.22 ± 3.32	82.73 ± 8.54	84.59 ± 8.06	79.26 ± 8.32
**Platelet k/μL**	341.69 ± 142.15	310.16 ± 123.8	395.04 ± 154.71	387.39 ± 141.79	368.86 ± 118.99	426.3 ± 128.23	273.37 ± 128.23	194.2 ± 43.11	361.33 ± 133.97	335.75 ± 127.39	304.54 ± 107.85	393.71 ± 139.99
**WBC k/μL**	10,042 ± 3741.03	9461 ± 3596	11,026 ± 3774	8922 ± 3228	8766 ± 3134	9250 ± 3049	10,205 ± 3049	9600 ± 2982	10,877 ± 2980	11,625 ± 4417	10,476 ± 4386	13,757 ± 3609
**Reticulo-cyte %**	11.04 ± 5.73	10.73 ± 5.61	11.56 ± 5.9	9.75 ± 5.6	10.15 ± 5.24	8.92 ± 5.24	12.41 ± 5.24	13.01 ± 6.25	11.76 ± 3.71	11.73 ± 5.96	9.92 ± 5.17	15.07 ± 5.87
**Total Bilirubin mg/dL**	3.42 ± 2.4	3.45 ± 2.73	3.38 ± 1.69	2.56 ± 1.52	2.51 ± 1.34	2.68 ± 3.19	4.61 ± 3.19	5.23 ± 4.03	3.91 ± 1.61	3.64 ± 2.07	3.61 ± 2.44	3.69 ± 1.12
**Ferritin ng/mL**	1251 ± 1744	1262 ± 1931	1230 ± 1249	526 ± 589	563 ± 653	401 ± 250	2752 ± 2258	3093 ± 2920	2374 ± 1013	717 ± 1156	929 ± 1300	167 ± 80
**LDH U/L**	451.65 ± 150.71	433.57 ± 161.02	482.27 ± 125.65	429.26 ± 139.62	416.5 ± 152.36	456.10 ± 103.03	442.68 ± 145.74	399.40 ± 141.56	490.8 ± 134.82	494.90 ± 162.43	487.46 ± 174.82	508.71 ± 135.40

Clinical and laboratory characteristics of all study participants divided by age and treatment group. ∞: defined as those on HU for a minimum of three months, α: patients on chronic transfusion therapy, ¥: therapy naive patients defined by lack of disease modifying therapies.

**Table 2:** Clinical laboratory values for BM samples, on hydroxyurea.

N = 5	Average	Standard Deviation
Years on HU	4.22	1.05
WBC k/μL	11.80	1.64
Hemoglobin g/dL	10.38	1.68
Hematocrit %	29.90	4.87
MCV fL	94.30	8.95
Platelet k/μL	407.50	90.93
Reticulocyte %	9.25	2.55
Hemoglobin F%	12.65	1.81
Hemoglobin S%	80.33	2.73
Hemoglobin A2%	4.17	0.79
Total Bilirubin mg/dL	2.55	0.50
LDH U/L	489.00	184.32
Ferritin ng/mL	313.00	136.57

## Supplementary Materials

The following are available online at https://www.mdpi.com/2073-4409/9/10/2159/s1, Figure S1: Clinical parameters, Figure S2: Antigen expression in CD34^dim^ cells, Figure S3: Reproducibility of the quantification of the HSPCs, Figure S4: Correlation between the percentage of HSPCs per CD34 cells in the peripheral blood and bone marrow, Figure S5: Concentrations of HPCs, CMPs, GMPs, and MEPs decrease as a function of length of HU treatment, Table S1: Antibodies.
