# Supplementary material for: Characterization of Hematopoiesis in Sickle Cell Disease by Prospective Isolation of Stem and Progenitor Cells
Source: Cells. 2020 Sep 24;9(10):2159. doi: 10.3390/cells9102159 (PMC7598721; doi:10.3390/cells9102159)
Supplement: Supplementary file 1 [file cells-09-02159-s001.pdf]

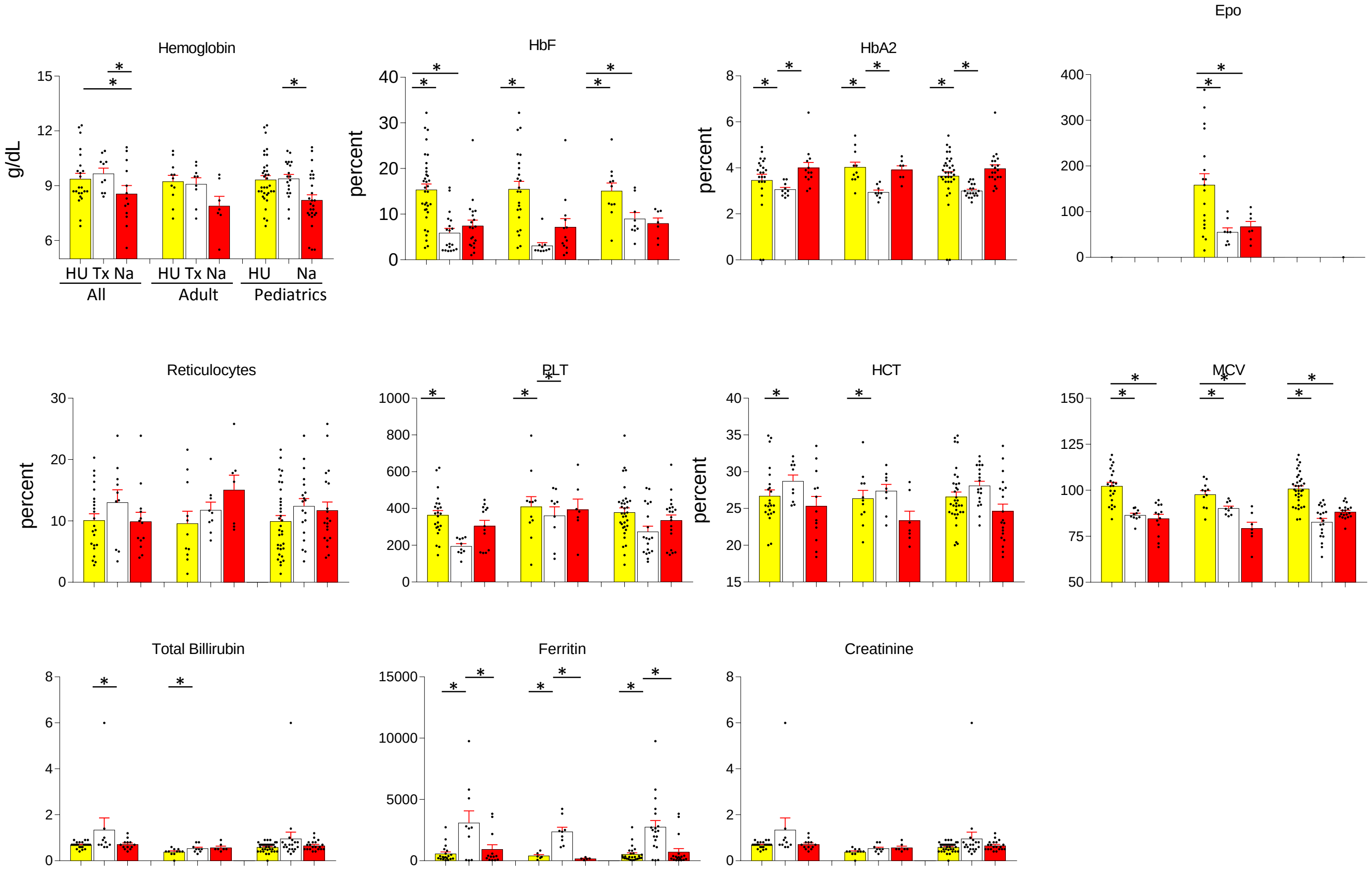

Figure S1: Clinical parameters: *Bargraphs illustrating Hemoglobin concentration, Hemoglobin F (Hb F), Hemoglobin A2 (Hb A2), Erythropoietin concentration (EPO), percentage reticulocytes, platelet concentration (PLT), hematocrit (HCT), Mean Corpuscular Volume (MCV), total bilirubin, ferritin and creatinine concentration. * = p-value < 0.05*

A

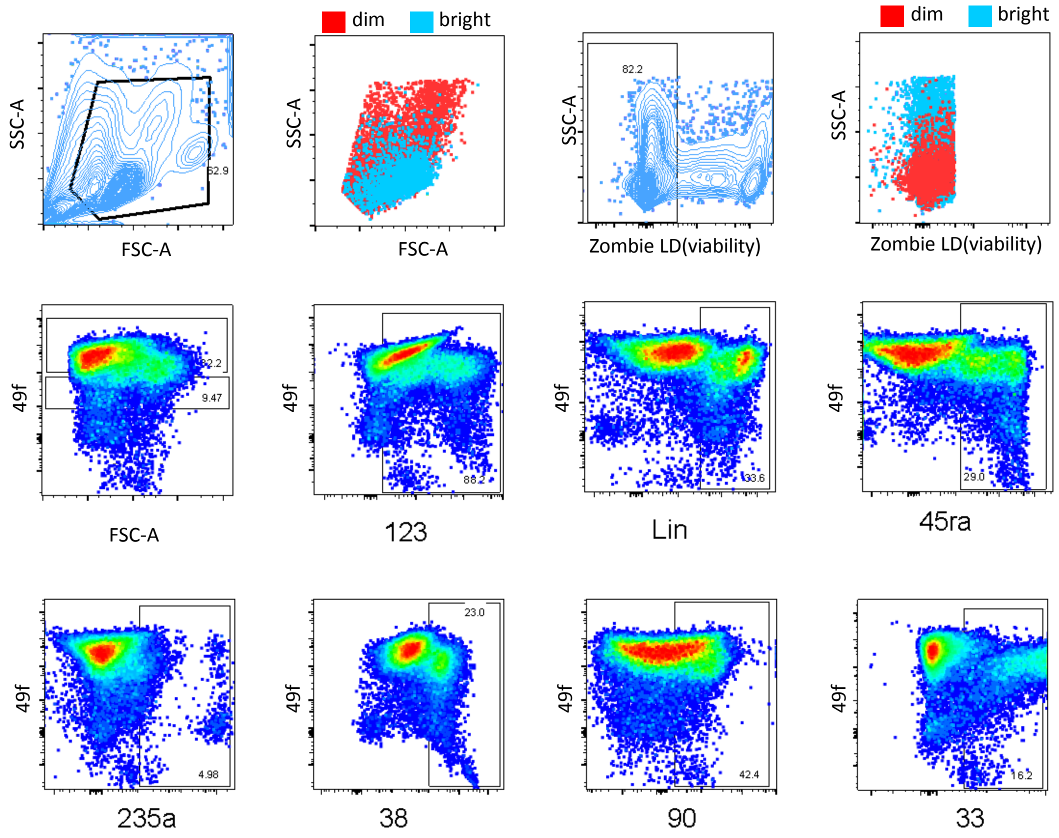

B

Status	n	CD123+	CD235a+	CD33+	CD38+	CD45RA+	CD49f	CD49f+	CD49f++	CD90+	Lineage+
**Healthy**	19	71.7 (3.35)	5.3 (1)	25.7 (3.92)	30.1 (5.9)	57.1 (4.81)	93.5 (0.32)	15 (2.3)	78.4 (16.32)	26.6 (2.82)	49.9 (4.89)
**HU**	29	90.2 (1.54)	8.2 (1.47)	26 (3.22)	36.1 (3.8)	44.1 (4.12)	84.9 (2.53)	13.8 (1.81)	71.1 (4.1)	47.7 (2.49)	46 (3.9)
**EX**	19	88.9 (2.35)	6.6 (1.64)	23.4 (4.31)	23.7 (4.58)	30.5 (4.92)	91.5 (2.13)	8.8 (1.7)	82.7 (2.58)	40.6 (3.36)	34.1 (5.42)
**Naive**	19	89.8 (1.32)	6.9 (2.47)	19 (2.42)	22.7 (3.07)	25.9 (3.32)	91 (2.92)	9.8 (1.83)	81.2 (3.31)	42.2 (2.21)	28.6 (3.1)

Figure S2: Antigen expression in CD34^{dim} cells. *A: Dot plot illustrating antigen expression of CD34dim cells in HU treated patients. Twenty nine FCS files were concatenated (10,000 cells per patients). The patterns of antigen expression for the healthy controls and the other SCD patients were similar.. The first row illustrates the scatter properties and the viability of the CD34dim cells (red) as compared to the bright cells (blue). Viable cells were defined as negative for staining with Zombie -green dye (LD Live/Dead dye). Rows two and three: All histograms represent the CD34dim cells. The rectangular gates outline the positive cells. The number inside the gates the proportion of positive cells relative to the total number of CD34dim cells. In the case of the 49f antigens, gates for CD49+ and CD49++ were drawn. B: Table illustrating the mean percentage (± SEM) of CD34dim cells expressing indicated antigens. The CD34dim cells express very high levels of CD49f and of CD123 and variable amount of other antigens.*

Reproducibility

relative to CD34+ cells

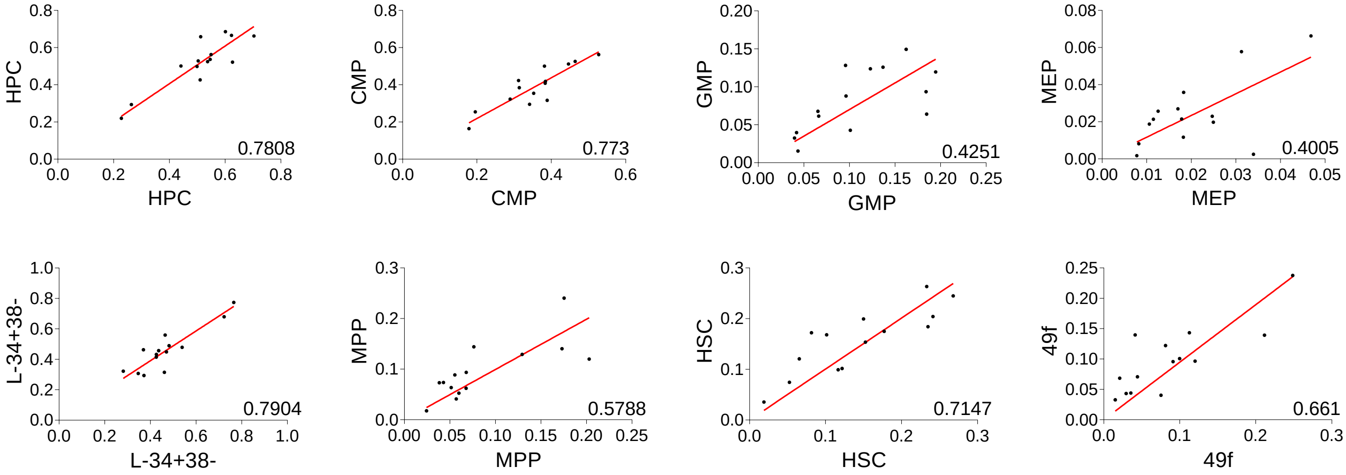

relative to total live MNC cells

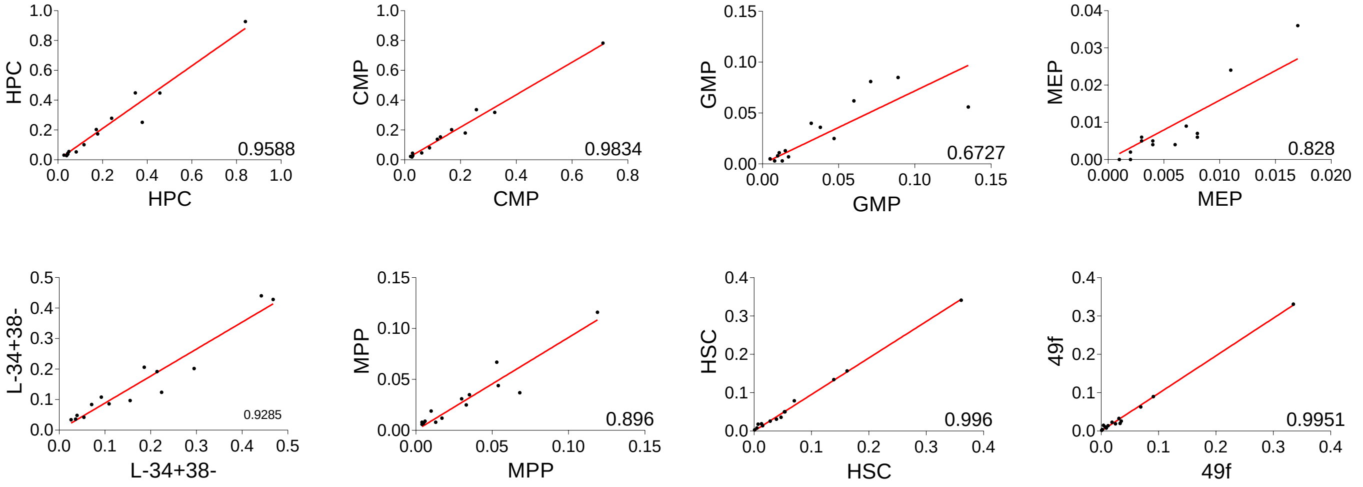

Figure S3: Reproducibility of the quantification of the HSPCs. *Frozen vials of mononuclear cells from fourteen individuals were thawed at two weeks intervals and the percentage of HSPCs was analyzed by FACS on a Cytek aurora. Data was then processed using FloJo in an automated manner. Plots represent regression analysis of these technical repeats after normalizing the results either to the number of CD34bright cells or to total live cells. In most cases, the reproducibility was very high. The few cases where reproducibility is lower (MEP for instance) was most likely caused by sampling error (very low cell numbers).*

A

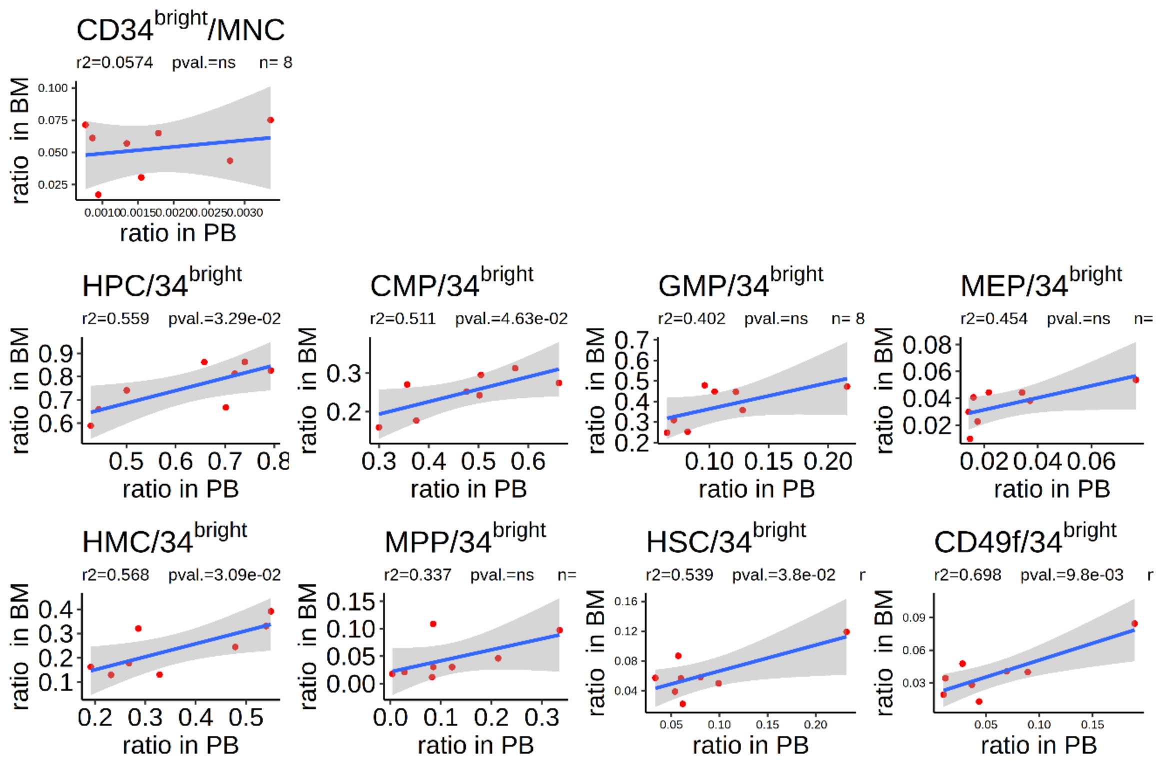

B

ID	Tissue	lin-	HMC	HSC	49f	MPP	HPC	CMP	MEP	GMP
**L1**	PB	95.26%	19.18%	3.35%	1.20%	8.49%	79.27%	66.15%	1.75%	10.45%
**L10**	PB	85.35%	26.74%	8.04%	6.95%	2.76%	71.98%	50.19%	7.66%	12.23%
**L11**	PB	89.28%	54.91%	9.92%	8.92%	33.47%	42.79%	29.96%	3.71%	7.01%
**L16**	PB	86.65%	28.61%	5.72%	3.68%	8.45%	70.16%	57.36%	3.41%	8.17%
**L2**	PB	88.31%	47.85%	5.39%	1.02%	21.32%	50.06%	37.46%	1.47%	9.61%
**L7**	PB	86.73%	32.84%	6.18%	4.35%	8.24%	65.79%	50.46%	2.17%	12.81%
**L8**	PB	91.69%	53.97%	23.20%	18.94%	12.20%	44.39%	35.63%	1.42%	6.44%
**L9**	PB	65.60%	23.20%	6.00%	2.80%	0.40%	74.00%	47.60%	1.60%	21.60%
**L1**	BM	79.32%	16.28%	5.73%	3.44%	3.01%	82.61%	27.45%	2.26%	44.84%
**L10**	BM	70.62%	17.84%	5.83%	4.07%	2.10%	81.25%	24.24%	5.37%	44.71%
**L11**	BM	79.41%	39.14%	5.00%	4.00%	9.74%	58.87%	15.94%	3.81%	30.95%
**L16**	BM	88.92%	32.05%	8.69%	2.84%	10.88%	66.72%	31.29%	4.42%	25.31%
**L2**	BM	81.02%	24.48%	3.89%	1.94%	4.61%	74.07%	17.71%	0.97%	47.88%
**L7**	BM	81.07%	13.09%	2.23%	1.31%	1.18%	86.22%	29.56%	4.43%	35.82%
**L8**	BM	86.19%	33.09%	11.91%	8.44%	3.01%	65.95%	27.07%	2.98%	24.86%
**L9**	BM	74.11%	13.01%	5.67%	4.77%	1.77%	86.32%	25.24%	4.06%	47.26%
**ratio BM to PB**		0.93	0.66	0.72	0.64	0.38	1.21	0.53	1.22	3.42

Figure S4: Correlation between the percentage of HSPCs per CD34 cells in the peripheral blood and bone marrow: *A: Peripheral blood and bone marrow samples were collected on the same day from eight healthy individuals and the percentage of HSPCs was determined. This graph illustrate the relatively strong correlation between the two measurements suggesting that the percentage of HPSCs measured in the peripheral blood reflects, in part, the atmosphere of the bone marrow. B: Table summarizing the results of the comparison between PB and BM HSPCs. The ratio between the proportion of HSPCs in the PB and BM was between generally 0.5 and 2 except for GMPs which were much more frequent in the BM than in the PB blood.*

Year on HU versus HSPC/mL of Blood (log transformed)

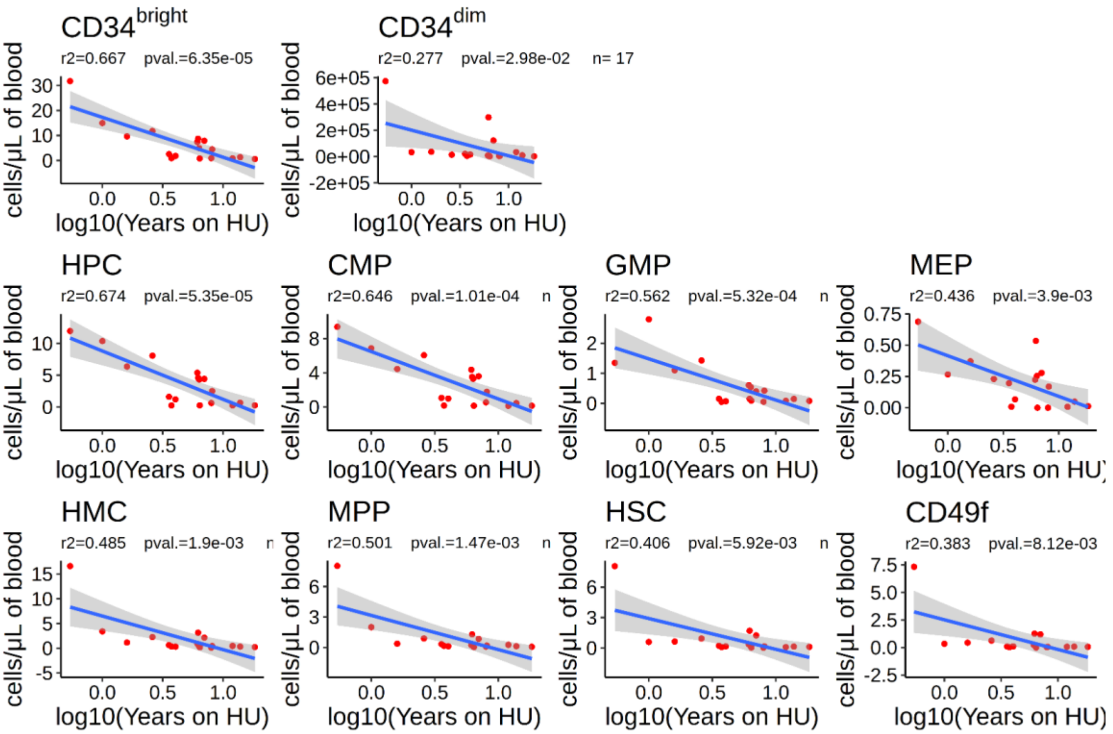

Figure S5: Concentrations of HSPC decrease as a function of the log of the length of HU treatment*. Linear regression illustrating the proportion of HSPCs/uL of blood relative to the length of HU treatments. The concentration of CD34bright, HPCs, CMPs, GMPs and MEPs relative to the length of time of HU treatment decreases over time. By contrast, the concentration of the late progenitors (CD34dim) and of the cells in the stem cell compartment (HMCs, MPPs, HSCS and 49f) exhibit less significant time dependency. (r2 and p-values for the linear regression is provided above each graph). Grey smooth represents the 95% confidence for prediction for the model. r2 and p-values were calculated after log-transformation of the x-axis.*
